# Interactions between commercial fishing vessels and a pelagic seabird in the southern Mediterranean Sea

**DOI:** 10.1186/s12898-018-0212-x

**Published:** 2018-12-04

**Authors:** M. Cianchetti-Benedetti, G. Dell’Omo, T. Russo, C. Catoni, P. Quillfeldt

**Affiliations:** 1 Department of Animal Ecology and Systematics, Justus Liebig University Giessen, Heinrich-Buff-Ring 26, 35392 Giessen, Germany; 2 Ornis italica, Piazza Crati, 15, 00199 Rome, Italy; 3 Laboratory of Experimental Ecology and Aquaculture, Dept. of Biology, University of Rome Tor Vergata, via Cracovia snc, 00133 Rome, Italy

**Keywords:** Fisheries discards, Fishing vessel-seabird interactions, Foraging ecology, Scopoli’s shearwater (*Calonectris diomedea)*, GPS, Accelerometer, Vessel Monitoring System (VMS)

## Abstract

**Background:**

Fishing activities can influence foraging behaviour of many seabird species worldwide. Seabirds are attracted by fishing vessels which can facilitate access to demersal fish as a novel food resource that otherwise would be unavailable. On the other hand, intense fishing activities cause depletion of fish stocks with a reduction of natural prey available for seabirds. Moreover, fisheries discards can have lower nutritional value than natural prey. However, the importance of fisheries discard for seabirds and the possible implications on their foraging ecology is still poorly understood. In this study, we analysed the interactions of Scopoli’s shearwaters (*Calonectris diomedea*) during their foraging trips with fishing vessels. We combined the GPS and accelerometer data of shearwaters with the GPS data gathered during the same period from fishing vessels. Accelerometers allowed us to identify the main behaviours of birds.

**Results:**

The presence of fishing vessels significantly affected the individual behaviour of Scopoli’s shearwaters. Birds increased the time spent sitting on the water within 1.28 ± 0.13 km of fishing vessels likely feeding or waiting for discards. Approaches towards vessels within the interaction distance were therefore classified as an interaction and were recorded in about 40% of individuals. Birds interacting with fisheries had longer flight time during their foraging trips and covered longer distances to reach more distant foraging areas compared with individuals not approaching vessels.

**Conclusions:**

Our results suggested that fisheries discard consumption might not be a profitable source of food for Scopoli’s shearwaters. Despite the high density of fishing vessels in the home range of Scopoli’s shearwater, most individuals did not interact with them. Accordingly, scavenging individuals showed a lower foraging efficiency than their conspecifics. Intraspecific competition for foraging areas might play an important role for the foraging decision of birds to consume fisheries discards.

## Background

Commercial fisheries produce worldwide 7.3 million tonnes of discards per year [1] which cause alterations of marine food webs. Seabirds are attracted by fishing vessels since fisheries discards represent a predictable and abundant source of food [2, 3] likely easier to obtain than natural prey [4]. Furthermore, fishing vessels make demersal fish available as a novel food source for seabirds, that would be unavailable otherwise [5, 6]. Thus, fisheries discards can represent an important source of additional food in the seabird diet [7, 8]. However, fisheries discards raise important conservation issues for seabirds [6, 9]. Interactions between birds and fishing vessels cause the accidental killing of thousands of seabirds every year, especially albatrosses and petrels [10–12]. Uncontrolled fishing can also cause a drastic reduction of natural prey availability for seabirds [5], followed by population reduction in some species [13, 14]. Moreover, fisheries discards can be a “junk food” since they are less nutritious compared with the natural prey of seabirds [15].

Given these multiple effects it is still not clear if seabirds benefit from fisheries discards and how discard consumption affects individual birds. This lack of information might be due to methodological limits to identify single interactions between birds and vessels. Methods calculating the overlap of the foraging areas of seabirds with the spatial distribution of vessels [16–18] might overestimate the use of fisheries discard by birds since seabirds can forage in the same areas where fishing vessels operate, albeit without interacting with them. Only few studies have analysed single interactions between birds and vessels. Specifically, the estimation of interaction distance was used to investigate how gannets and albatrosses change their behaviour according with the presence of fishing vessels [19, 20]. The identification of single interactions allow researchers to assess the importance of fisheries discards for seabirds populations and individuals and thus to plan effective conservation actions.

In this study, we identified the interactions of Scopoli’s shearwaters (*Calonectris diomedea*) with fishing vessels and assessed the effects on the foraging ecology on these seabirds. The study was carried out on Linosa Island, located in the Sicily Channel in the central Mediterranean Sea, an area intensively exploited by fishing vessels [1, 21]. Scopoli’s shearwaters lay a single egg per breeding season in the second half of May to hatch in mid-July. Parental duties are shared between parents. During incubation, parental birds alternate long fasting periods with foraging trips lasting several days [22]. During chick rearing, parental birds need to attend the nest frequently, especially at the beginning, performing short foraging trips close to the colony [23].

Scopoli’s shearwaters consume a wide range of prey [24] including fisheries discards [25, 26]. In order to identify interactions we used the spatio-temporal positions of both fishing vessels and Scopoli’s shearwaters to assess when each bird actively followed a certain fishing vessel (approaching events). In addition, we estimated the interaction distance between birds and fishing vessels [20] through the analysis of the time-budget of birds’ behaviour, derived from accelerometer data. Then, we tested if the occurrence of one or more interactions affected the individual foraging behaviour of birds, and in particular: (1) the daily energy spent, (2) the time spent flying, (3) the maximum linear distance reached from the colony and (4) the total trip length of foraging trips.

## Results

**Fig. 1:**
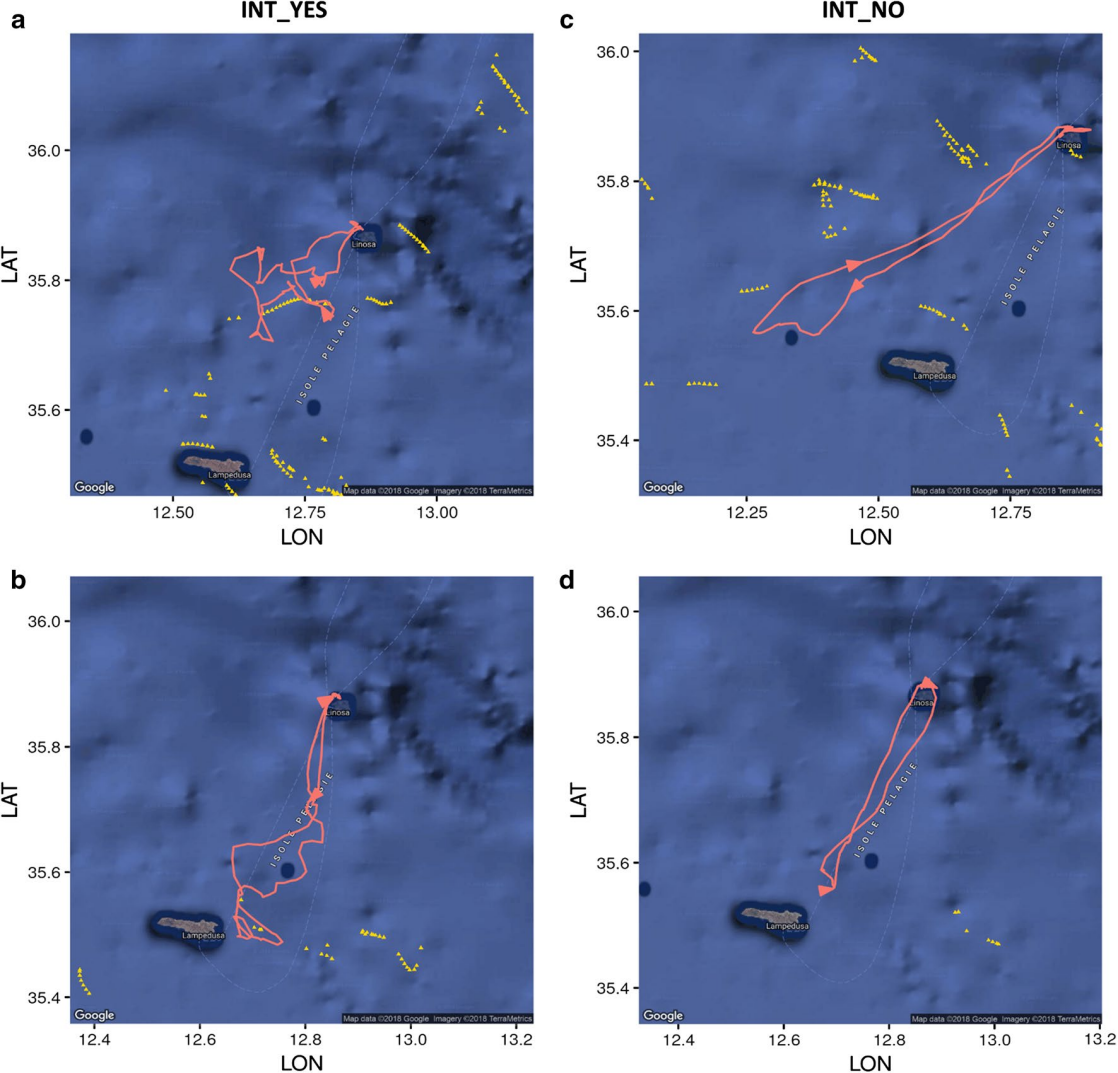
Examples of short foraging trips performed by Scopoli’s shearwaters during chick rearing (red lines) in relation to the presence of fishing vessels (yellow triangles). INT-YES (**a**, **b**): individuals interacted with fishing vessels; INT_NO (**c**, **d**): individuals did not interact with fishing vessels. The direction of the bird during each foraging trip is indicated with an arrow

We recorded a total of 206 (30 long, 176 short) complete foraging trips with a maximum duration of 13 days. In 160 of these we recorded at least one interaction event (63.5%; Fig. 1). A total of 414 fishing vessels were operating in the central-south Mediterranean Sea within the home-range of Scopoli’s shearwater from Linosa Island during the study period. Most of the fishing vessels were trawlers (76%), while longlines, purse seine vessels and unknown fishing vessels amounted on 6%, 11% and 7% respectively (Table 1). Birds interacted more frequently with trawlers (87%) compared with other fishing vessels, such as longline and purse seine vessels (χ^2^ = 8.19, df = 1, p < 0.004). No sexual difference in frequency of interaction with fishing vessels were observed (χ^2^ = 0.03, df = 1, p > 0.05).

**Table 1:** Types of fishing vessels operating in the Strait of Sicily during the breeding period of Scopoli’s shearwater (from 6th June to 20th August)

Gear/type of fishing activity	Number of vessels
Bottom Otter trawl	315
Purse Seine	45
Longline	25
Other Gears	29
Total	414

**Fig. 2:**
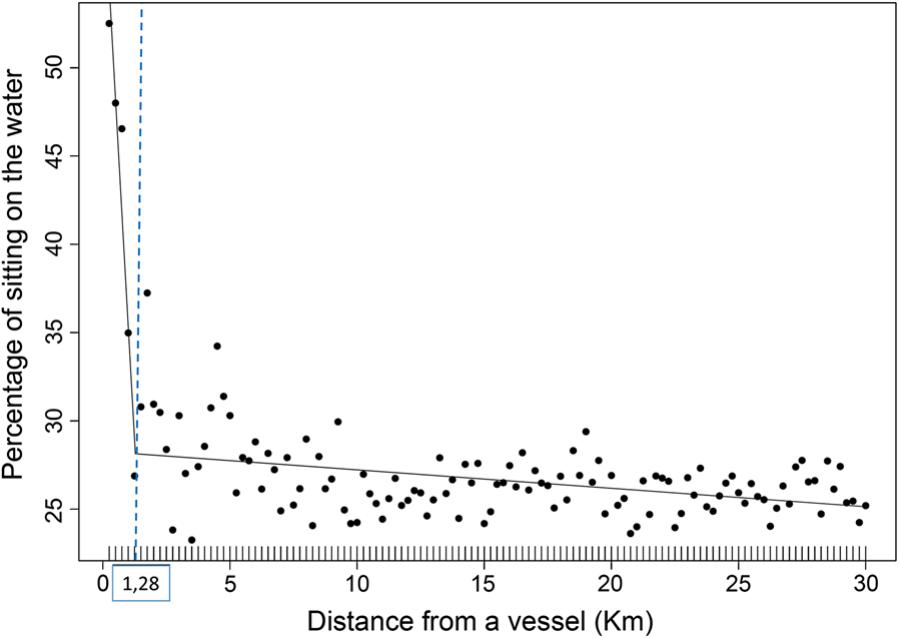
Percentage of “sitting on the water” behaviour of Scopoli’s shearwaters in relation to their distance from fishing vessels. Each point in the graph represents the average percentage of “sitting on the water” calculated from > 110 bird-vessel positions corresponding to a “distance from a vessel” bin of 0.25 km. Two-part piecewise linear regression was used to estimate the “interaction distance” resulting at 1.28 km (95% CI 1.15–1.43 km), as indicated by the dashed line

**Fig. 3:**
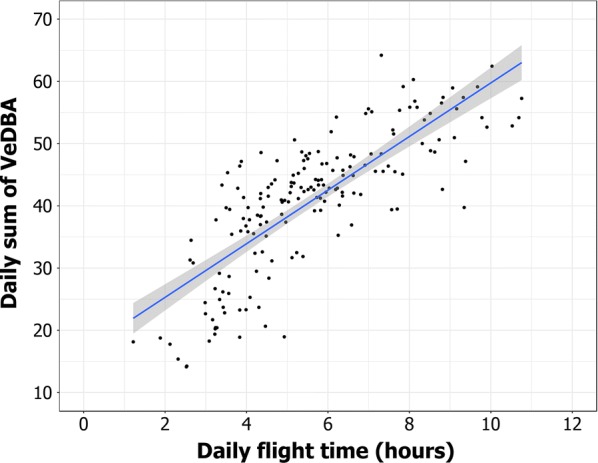
Relationship between the “Daily sum of VeDBA” and “Daily flight time” calculated during short foraging trips of Scopoli’s shearwaters during chick rearing

We identified a total of 232 “interaction events” in the period of study. Of 75 tracked individuals, 31 (41%) interacted at least once with a fishing vessel. Slightly fewer interactions were recorded during short foraging trips (38%). Birds spent most of their time sitting on the water within 1.28 ± 0.13 km from a fishing vessel (interaction distance). Beyond this “interaction distance” the time spent by birds sitting on the water sharply decreased followed by more constant, lower values when their distance from fishing vessels increased (Fig. 2). The “interaction distance” was calculated using a high number of occurrences (> 4500 distance points between birds and fishing vessels) so as to reduce the possible error due to the GPS positions used for birds and fishing vessels and individual differences in behaviour. We found a positive linear relationship between “daily flight time” and “daily sum of VeDBA” (F_1,174_ = 321.4, p < 0.001; Fig. 3). This means that flight behaviour is energetically expensive for these seabirds. Indeed, the “daily sum of VeDBA” represents a good proxy of the relative energy spent by an individual: high values of VeDBA corresponded to high activity of the birds (e.g. long flight time). We tested for the effects of (1) sex and (2) the occurrence of one or more interaction events (INT) on several dependent variables determining the foraging behaviour during short foraging trips. Birds spent daily more energy (VeDBA) in trips with at least one interaction (INT-YES) compared with trips without interactions (INT-NO; F_1,124_ = 14.65, p < 0.001). Furthermore, the INT-YES birds spent daily more time flying (F_1,124_ = 10.28, p < 0.002), performed longer foraging trips (F_1,124_ = 10.68, p < 0.002) and flew farther from the colony (F_1,124_ = 12.08, p < 0.001) than INT-NO (Table 2).

**Table 2:** Averages (± SD) of foraging variables in Scopoli’s shearwaters between (1) individuals that interacted with a fishing vessel at least one time (INT-YES) and (2) seabirds that never interacted (INT-NO) during a foraging trip

	Interaction (INT-YES)	No interaction (INT-NO)
Daily sum of VeDBA	44.77 ± 9.78	38.63 ± 11.07
Daily flight time (hours)	6.22 ± 1.98	5.28 ± 1.96
Max linear distance from the colony (km)	74.54 ± 44.19	53.72 ± 38.59
Trip length (km)	375.02 ± 222.91	243.27 ± 136.76

**Fig. 4:**
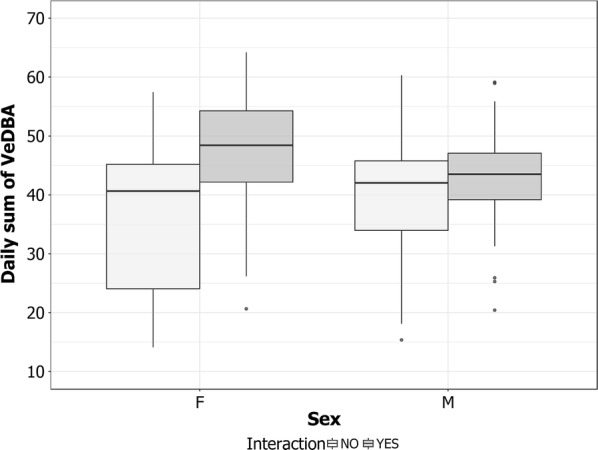
Differences of “Daily sum of VeDBA” in relation to sex and occurrence of interaction of Scopoli’s shearwaters with fishing vessels: INT-YES (individuals have interacted with fishing vessels), INT-NO (individuals that did not interact with fishing vessels)

We did not find any differences between sexes regarding VeDBA (F_1,48_ = 0.01, p > 0.05), time spent in flight (F_1,48_ = 0.52, p > 0.05), foraging trip length (F_1,48_ = 1.31, p > 0.05) or maximum distance reached by birds from the colony (F_1,48_ = 0.22, p > 0.05) during foraging trips. However, females spent more energy than males when interacting with fishing vessels (F_1,124_ = 4.08, p = 0.045; Fig. 4).

## Discussion

Our study aimed to identify the individual interactions of Scopoli’s shearwaters with fishing vessels operating in the Mediterranean Sea. We combined spatio-temporal data from birds during the reproductive period and fishing vessels to assess interactions. Then, we investigated the effects of these interactions on the foraging behaviour of the shearwaters.

The presence of fishing vessels significantly affected the behaviour of shearwaters. Birds spent significantly more time sitting on the water within 1.28 ± 0.13 km from a fishing vessel (Fig. 2), likely feeding or waiting for discards [20]. This interaction distance is less than half of the feeding distance found in an albatross species (3 km, [20]). The interspecific variation of the interaction distance might be explained by different factors such as the different foraging strategy of shearwaters and albatrosses (greater wing surface areas of albatrosses might be unfavourable to frequent take offs, bird densities in foraging areas and different environmental conditions such as natural prey availability, [27]). Other studies on interactions between shearwaters and fishing vessels did not include the interaction distance. However, it was observed that Scopoli’s shearwaters adjust their movement pattern according with the presence of fishing vessels [7], confirming the importance of fisheries discards at least for some populations. Among Mediterranean shearwaters, fisheries discards represent an important source of food for the threatened Balearic shearwater (*Puffinus mauretanicus*) [8, 9], specifically during the breeding season [2] which can improve the breeding performance of this species [28].

The type of vessel might also have influenced the interaction distance. In the study area of albatrosses [20], seven long-liners and no other vessels type were operating. However, in our study area mostly trawlers occurred, and Scopoli’s shearwaters interacted more frequently with trawlers compared to other types of vessels available in their home-range. This species might prefer this fishing vessel type since trawlers produce high amounts of discards [29] characterized by a large array of species [30] including the main prey of shearwaters [28].

**Fig. 5:**
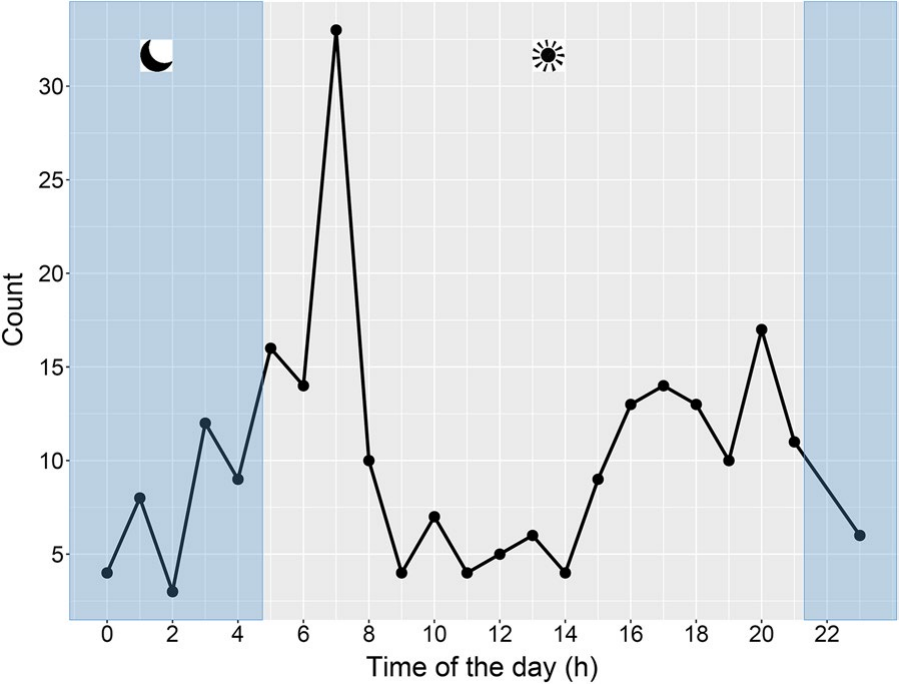
Number of interactions between birds and fishing vessels calculated per time of the day (GMT +2). The grey area indicates the night hours

The number of interactions performed by the birds significantly changed over the time of the day: the highest number of interactions were observed in the early morning, about 2 h after parents left the colony. The shearwaters interacted with fishing vessels mainly during the first part of their foraging trip, likely in order to self-feed and thus recover after chick provisioning [31]. During the central part of the day, the number of interaction drastically decreased to increase again in the late afternoon (Fig. 5). During night time birds still interacted with fishing vessels but less frequently than during the day (Fig. 5).

**Fig. 6:**
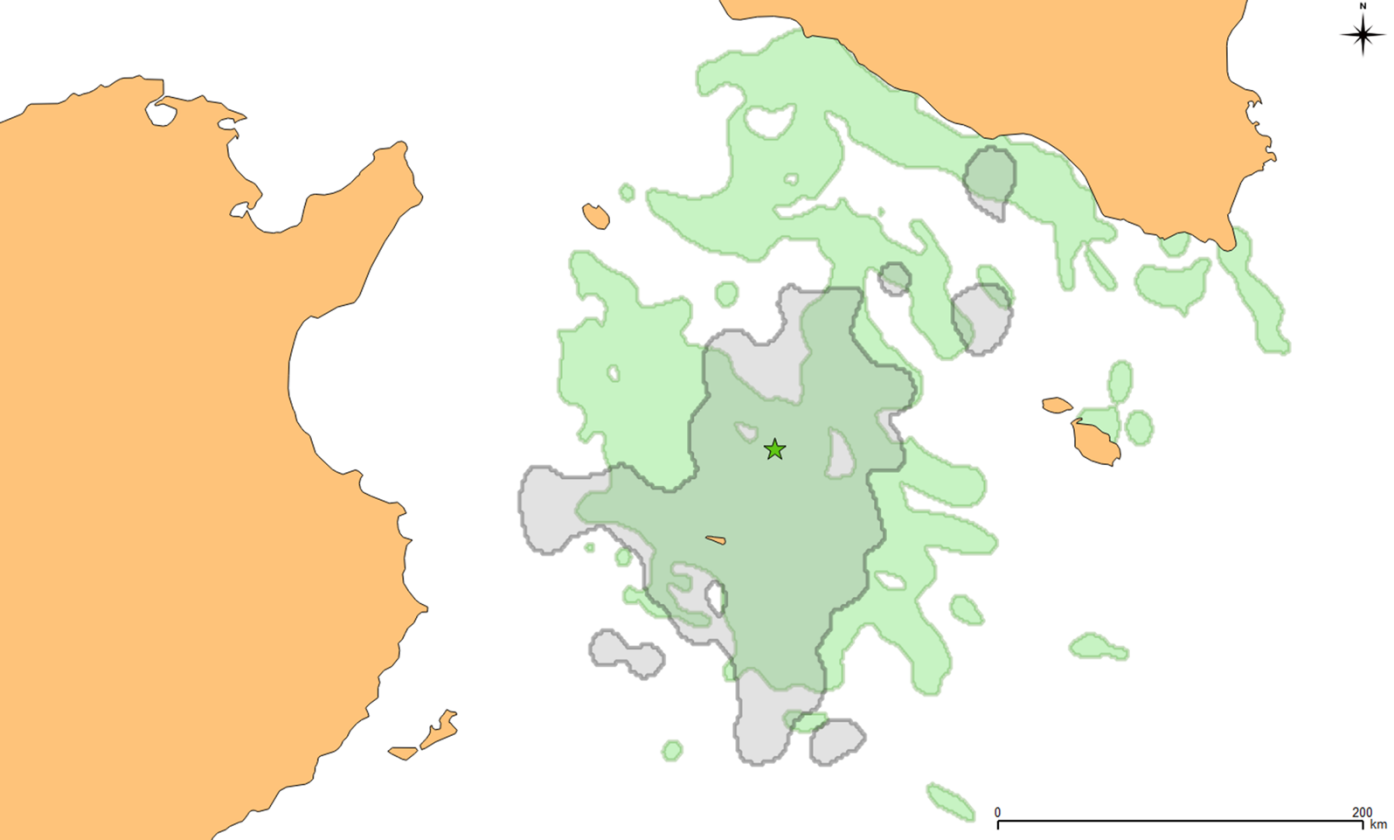
Home range of short foraging trips (≤ 3 days) of Scopoli’s shearwaters (grey) and operating area of fishing vessels (green) with 95% kernel UD during the chick-rearing seasons of 2016

The central-south Mediterranean area hosts the largest colonies of Scopoli’s shearwaters (Zembra and Linosa Islands). At the same time, hundreds of fishing vessels operate in this area [1, 21]. These intense fishing activities cause overexploitation of fish stocks with a consequent reduction of prey availability to sustain such a high number of seabirds [5]. On the other hand, fishing vessels produce large amounts of discards which can potentially represent an additional food resource for seabirds [5, 6]. Considering only the home-range of Scopoli’s shearwater during short trips, we observed that this widely overlapped with areas where fishing vessels operate (Fig. 6). Consequently, the probability for a bird to encounter a fishing vessel was reasonably high. Therefore, we expected that shearwaters use fisheries discards extensively as a predictable food resource [7]. However, a considerable proportion of individuals never interacted with any fishing vessels during short foraging trips (62%). This result suggests that most of the Scopoli’s shearwaters breeding on Linosa Island might not directly benefit from exploiting discards, but preferred taking natural prey instead. Accordingly, other studies observed that fisheries discards were not the major part of the diet in other seabirds [4, 32]. The relatively low proportion of seabirds following fishing vessels can be explained considering that fisheries discards can have lower caloric values compared to the natural prey of seabirds (“*Junk food hypothesis*”, [15]). For this reason, natural prey might be preferred by seabirds during high energy demanding phases such as chick rearing [33] and during short foraging trips, when the parents increase their foraging effort [23]. However, given that this species is considered a flexible forager [24], from the present data we cannot exclude the possibility that the proportion of scavenging birds can significantly change according to the breeding stage or the availability of their natural prey in different years. Further studies are needed to understand which physiological and environmental factors influence the decision of seabirds to consume fisheries discards.

Despite the fact that a large proportion of birds in this study never interacted with a fishing vessel, fisheries discards can be an important component in the diet of certain individuals [4] influencing their foraging behaviour [7, 19]. Our data showed that individuals that never interacted with vessels (non-scavengers) remained closer to the colony compared with birds that attended a vessel at least one time (scavengers). Moreover, non-scavengers spent less energy due to shorter flight times and distances covered per foraging trip. This result suggests that competitive exclusion might drive the foraging decision of birds to attend fishing vessels. Scopoli’s shearwaters from Linosa Island performed short foraging trips mostly in sub-optimal foraging areas surrounding the colony [22, 34]. Therefore, this area can be easily overexploited leading to a fast depletion of natural prey availability [35]. As a consequence, only more competitive birds might have access to foraging areas surrounding the colony without using discards. Conversely, less competitive birds may be forced to forage further away from the colony where competitive interactions may be reduced [36]. These individuals might consistently use fisheries discards to cover the additional energy cost due to prolonged flight time needed to reach more distant foraging areas.

Both sexes interacted with fishing vessels with the same frequency, similar to observations in two albatross species [32]. However, females spent more energy than males during foraging trips but only when they interacted with fishing vessels. Nevertheless, no differences in flight time, distance covered and linear distance from the colony reached during foraging trips were recorded between males and females independently of the occurrence of an interaction. Scopoli’s shearwater is a dimorphic species where males are larger than females in body mass and wing surface [37]. Larger males were observed to be more competitive than females [36]. Seabirds attend the fishing vessels mainly sitting on the water waiting for discards [20] often in large numbers which might cause high competition [38]. In these conditions males might be more competitive than females when they interact with fishing vessels, forcing the females to take off more frequently than males and resulting in higher energy consumption. However, further investigations are necessary to better understand behavioural differences between sexes in discard exploitation.

## Conclusion

The identification of seabird–fishing vessel interactions using high resolution devices such as GPS and accelerometers can effectively contribute to understand the effects of fisheries discards on the foraging ecology of seabirds. It is urgent to increase our knowledge about this topic since the European Union’s Common Fisheries Policy aims to ban fisheries discards in 2020. The possible consequences of this action for seabird communities are still poorly understood.

This study was carried out in the central-south Mediterranean Sea, an area intensively exploited by hundreds of fishing vessels, which can be considered a valid case study to assess the seabirds’ response to overfishing conditions and marine stock depletion. Despite the high density of fishing vessels operating in the home-range of Scopoli’s shearwaters breeding in Linosa Island, most of the tracked birds never interacted with the vessels. This might indicate that these shearwaters prefer to exploit natural prey even if they are less predictable and more difficult to obtain than fisheries discards. However, about 40% of birds interacted at least one time with a fishing vessel suggesting that natural prey availability might be insufficient to sustain the energy requirements of the entire population of Scopoli’s shearwaters breeding at Linosa Island.

Our study suggests that abrupt banishment of discards may cause an ecological disturbance to this population of Scopoli’s shearwaters. Thus, the elaboration of a gradual reduction plan of discards by the fisheries authorities would be strongly recommended in order to monitor the effects of changes of discard availability on the foraging ecology of this population.

## Methods

### Data collection

The study was carried out during the breeding season 2016 (from June 6th to August 20th) in a colony of Scopoli’s shearwaters located on Linosa Island (35°51′33″N; 12°51′34″E). The colony site extends along the northern part of the island and hosts about 10,000 breeding pairs of Scopoli’s shearwaters [39] resulting in the largest European breeding colony of this species. Scopoli’s shearwaters breed mostly in crevices of the lava rocks.

We tracked 75 birds with Axy-trek dataloggers (Technosmart Europe S.r.l), including 12 birds during incubation and 63 during chick rearing. The loggers include a tri-axial accelerometer, a GPS, a thermometer, and a TDR (Time Depth Recorder). The GPS was set to record positions every 5 min. The accelerometer was set at 25 Hz and the TDR recorded pressure at 1 Hz with an accuracy of 5 mBar (≈ 0.05 m). Data from TDR and temperature were not used in this study.

Birds were captured at the nest and a device was attached on the back feathers using marine waterproof Tesa^®^tape [40]. Birds were sexed according to bill measurements [41] and vocalizations. The handling during the procedure did not take longer than 10 min. Afterwards, the birds were released into the nest, and they were recaptured after a minimum time of 10 days (range 10–13 days) for retrieving the device. The device was carefully removed from feathers together with the tape and its residuals. We did not observe any nest desertion due to our operations.

### Fishing vessels data

The positions of Italian fishing vessels active in the central southern Mediterranean Sea (Strait of Sicily) were reconstructed using the data provided by the Vessel Monitoring System (VMS). These data were made available from the Italian Ministry of Agricultural, Alimentary and Forestry Politics, within the activity for the Italian National Program for the Data Collection in the Fisheries Sector. The VMS, introduced by the European Union for enforcement on fishing activity [42, 43], consists of an automatic transmitting station called “blue-box”, which periodically sends information (“ping”) about GPS vessel position, speed, and prow heading [43] through a satellite network. The VMS data for Italian fishing vessels with length ≥ 15 m and operating in the study were processed using the platform VMSbase [44]: basically, VMS pings were organized in separated fishing trips by vessel, interpolated from the original frequency (one ping every 1–2 h) to a higher value (1 ping every 10 min) to better reconstruct vessels paths at sea [45], and classified in terms of gear used (with trawling, purse seining and longlining being the main typologies of fishing gear) and of activity (steaming/fishing) [46]. The interpolation procedure merits particular attention since it returns pings aligned to a specific time scale, as if all the vessels had sent their signals simultaneously (whereas the native pings are randomly distributed in time), so that the user can get a ‘‘snapshot’’ of all the vessel’s positions at any given instant of time. The distance between estimated and real positions is 0.65 (mean) ± 0.22 (standard deviation) km for trawlers and 0.81 ± 0.28 km for purse seiners and longlines [45].

At the end of this processing procedure, the activity of 414 fishing vessels operating in the central-south Mediterranean Sea within the home-range of Scopoli’s shearwater from Linosa Island during the study period was obtained as series of pings classified as steaming or fishing by means of speed filters, a standard approach for this purpose [46]. Each ping corresponded to information about spatial and temporal coordinates, type of fishing gear, and course of the vessel. This dataset was submitted to the successive analysis.

### Behavioural data analysis

Accelerometer data were used to identify three behaviours of birds: “sitting on the water”, “flapping flight” and “gliding flight”. The accelerometer recorded the acceleration in three axes corresponding with the bird orientation: X (head–tail), Y (right–left) and Z (dorso-ventral). In order to identify “sitting on the water” behaviour, Vectorial Dynamic Body Acceleration was calculated $\left( {VeDBA \left( g \right) = \sqrt {a^{2}_{x} + a^{2}_{y} + a^{2}_{z} } } \right)$ with 1 s smoothing [47]. The terms a_x_, a_y_ and a_z_ are the values of dynamic acceleration from X, Y and Z axis, respectively [47]. Dynamic Body Acceleration is a good proxy for estimating of energy expenditure [48, 49] where low values correspond to low activity of the individual. We assigned “sitting on the water” behaviour when the value of VeDBA was ≥ 0.1 and ≤ 1 using the birds ground speed calculated by GPS logger and by visual checking of GPS tracks of 30 birds.

“Flapping flight” behaviour was identified by the analysis of the absolute value of the Z axis at 25 Hz using Ethographer (version 2.03, [50]) an Igor-Pro extension (WaveMetrics, Version 6.05): spectrum analysis allowed us to calculate two clusters where the one with the higher cycle amplitude peak represented the flapping behaviour. The “gliding flight” behaviour was identified using the interval between two consecutive flapping events. In fact, the flight behaviour of Scopoli’s shearwater is an alternation of flapping and gliding events. For this study we used the daily sums of “flapping flight” and “gliding flight” time per individual, which was named “Daily flight time”.

### Assessing bird–fishing vessels interaction

In order to determine bird–fishing vessel interactions we used a two-step procedure. Firstly, we identified when a bird flew towards a vessel (approaching phase). Secondly, we calculated the distance at which birds interacted with fishing vessels feeding or waiting for discards (interaction distance, [20]). The approaching phase was determined by combining GPS data from birds and fishing vessels: (1) for each GPS bird position, we selected all the VMS data in the temporal window *t*_-*5′*_–*t*_+*5′*_ (where *t* is the temporal coordinate of the bird position). Notice that this approach guarantees that one single ping for each vessel was selected and associated to a bird position; (2) the linear distance from the bird position and all the vessels in a range of 50 km was calculated; (3) if at least one vessel was present in the range of 50 km, the previous step was repeated for the time *t*_+*1*_, *t*_+*2*_ and so on, in order to obtain a series successive distances between each bird and vessel; (4) then, for each bird, we filtered all the consecutive GPS positions (by time) where their distance from a definite fishing vessel gradually decreased by at least 500 m. These approaching events included at least two temporally consecutive GPS bird positions (> 10 min). The maximum attraction distance, which corresponded with the start of the approaching event, was set at 30 km [20]. However, it was evident that in many approaching events the birds stopped to follow the fishing vessels before reaching it.

As second step we assessed if a bird interacted with a fishing vessels by the estimation of the “interaction distance” [20]. While during the approaching phase the birds spent more time flying, they switched their behaviour when they were closer to fishing vessels. Specifically, the “sitting on the water” behaviour in the time budget showed a sharp increase followed by more steady, low values with increasing distance from a fishing vessel (Fig. 2). Thus, we used a piecewise linear regression [51] to calculate the threshold distance where birds spent significantly more time “sitting on the water” in response to a fishing vessel (interaction distance). For this purpose, the R package “Segmented” [52] was used to find the “interaction distance” with a binned distance of GPS bird point with fishing vessels set at 250 m (Fig. 2). Within this “interaction distance” we assumed that birds interacted with fishing vessels feeding or waiting for discards [20]. The “interaction distance” was used to validate the approaching events: only if a bird approaching a fishing vessel was observed to reach inside the range of the “interaction distance” it was considered as an “interaction event”.

### Data analysis

During the chick rearing phase most of the tracked birds performed multiple foraging trips. We determined the start and the end of different foraging trips and their duration using GPS positions of each bird. The “foraging trip length” and the “maximum linear distance reached from the colony” were then calculated from GPS data for each foraging trip.

We ran a linear regression in order to test the relationship between “Daily flight time” as dependent variable and “Daily sum of VeDBA” as covariate.

The number of interactions (interaction events) between birds and fishing vessels were calculated for each trip. Then, we defined a 2-level factor according to the number of interaction events recorded per foraging trip (INT: INT-NO = no interactions; INT-YES = number of interactions ≥ 1). We run four Linear Mixed Models (LMMs) using restricted maximum likelihood using the R-package *“nlme”* [53] to assess the effect of sex and INT on four dependent variables: (1) daily sum of VeDBA, (2) daily flight time, (3) trip length, and (4) maximum linear distance reached from the colony during a foraging trip, with bird identity as Random factor. Log-transformations were used when dependent variables were not normally distributed. Distribution, spatial autocorrelation and homoscedasticity of residuals were checked visually.

Given that our data did not include non-Italian fishing vessels, all LMMs were ran taking into account only the short foraging trips (up to 3 days) performed by birds during the chick rearing phase [23]. Indeed, Scopoli’s shearwaters of Linosa Island perform short foraging trips close to the colony [34] mostly in the Italian territorial waters. Chi-square tests were used in order to test (1) sex differences of interaction occurrences and (2) frequency of birds’ interactions in relation to the availability of different fishing vessel types. All statistical analyses were performed with R version 3.3.3 [54].

## Abbreviations

INT-YES: individuals interacted at least one time with fishing vessels during a foraging trip
INT-NO: individuals did not interacted with fishing vessels during a foraging trip
VeDBA: Vectorial Dynamic Body Acceleration
VMS: Vessel Monitoring System
